# Study of Room Temperature Ionic Liquids as Gas Sensing Materials in Quartz Crystal Microbalances

**DOI:** 10.3390/s20144026

**Published:** 2020-07-20

**Authors:** Manuel Aleixandre, Takamichi Nakamoto

**Affiliations:** Institute of Innovative Research, Tokyo Institute of Technology, Yokohama 226-8503, Japan; aleixandre.m.aa@m.titech.ac.jp

**Keywords:** gas sensor, room temperature ionic liquid, polar and non-polar, gas chromatography stationary phase, electronic nose, quartz crystal microbalance

## Abstract

Twenty-eight quartz crystal microbalance (QCM) sensors coated with different sensing films were tested and analyzed in this work; twenty-three sensors were coated in different room temperature ionic liquids (RTILs) and five additional QCM sensors were coated with conventional films commonly used as stationary phases in gas chromatography. Four volatile organic compounds (VOCs), in gaseous phase—hexanol, butyl acetate, 2-hexanone, and hexanoic acid—were measured. Two transducer mechanisms were used; resonant frequency shift and resistance shift of a QCM Mason equivalent circuit. The sensors were characterized by their sensitivity to the VOCs and their discrimination power of the four VOCs. The highest separation among VOCs was obtained when frequency and resistance information of both RTIL and conventional films was used, a sensor array composed by two RTILs (1-butyl-1-methylpyrrolidinium bis(trifluoromethanesulfonyl)imide and 1-hexyl-3-methylimidazolium hexafluorophosphate) and two conventional films (tricresyl phosphate and apiezon-L) was found to improve the Wilks lambda separation for the tested gases two orders of magnitude compared to the Wilks lambda using only a conventional films array.

## 1. Introduction

Electronic noses are devices that identify and quantify volatile compounds or odors [1]. They are composed of a gas sensor array, electronics to interrogate the sensors, algorithms to analyze the measurements data, and some means to acquire the sample gas. These devices are being studied to monitor atmospheric pollution [2], in the food industry [3], for health diagnosis [4], and for odor reproduction [5].

A very important part of the work made to improve the electronic noses is done by research in gas sensors, trying to improve the sensors in important characteristics such as selectivity, sensitivity, and stability [6], and other characteristics such as response time [7], sensor reproducibility [8], and cost [9]. Other developments on electronic noses try to improve the portability by miniaturization [10]. A very important part of the research is done by developing better automatic learning algorithms applied to electronic noses such as deep learning algorithms [11].

In electronic noses, it is a common practice to increase the number of sensors in the sensor array mimicking biological systems [12]. However, this approach has its limits, as the co-linearity of similar sensors can be high and decrease the performance. Because of this, many studies have shown that reducing the number of elements in sensor arrays improves the classification [13]. Other way to overcome this problem is to develop new sensor materials and configurations [14]. In this line of work, our group has studied electronic noses using an array of quartz crystal microbalance (QCM) sensors [15]. Although stationary phase materials for gas chromatography and lipids were used in previous studies [16,17], other kind of sensing materials are expected to enhance the selectivity.

Since QCM sensors coated with gas chromatographic stationary phases were used to detect gases [18], they have been used extensively as gas sensor elements in electronic noses [19]. As gas sensors, they have advantages such as low cost, commercial availability, easy manipulation, simplicity of the electronic circuits, and good sensor stability [19] compared to more complex devices such as surface acoustic waves (SAW) [20], thin film bulk acoustic resonators (FBAR) [21] or cantilevers [22] that usually have higher sensitivities. Some recent studies focused their research on the electronics by interrogating the sensors with several harmonics [23], employing diverse data treatments such as deep learning to link sensor data to human textual fragrance representation [24], or researching new sensing materials [25].

To increase the range of potential sensing materials available for building sensor arrays, this paper focuses on room temperature ionic liquids (RTILs) as gas sensing materials. RTILs are salts that remain liquid at room temperature [26] and in general have several advantages as gas sensor materials. They are stable and have very low volatility, factors that decrease the effects of drift or sensor poisoning. They remain liquid in a wide range of temperatures. Since they have a variety of combinations of cations with anions, they can be fine-tuned to interact differently with different gases. They have been studied as stationary phases in gas chromatography [27], sensing materials in electrochemical gas sensors [28], and in a few applications as sensor material coatings for QCM sensors [29].

To investigate RTILs as sensing materials, in this work, twenty-three RTILs were systematically studied to detect four odorant VOCs; some of these RTILs have never been used as gas sensors with QCMs. This is the first characterization of RTILs and gas chromatography stationary phases as sensing films for QCMs using the same procedure, testing every film in the same conditions so RTIL films and conventional films can be compared. To improve the classification, two different features were extracted from the sensors—resistance changes and resonance frequency shifts. Moreover, the discrimination capability using pure RTILs were compared with the discrimination power using pure gas chromatography stationary phases and with the discrimination power using a combination of RTIL and gas chromatography stationary phases.

## 2. Materials and Methods

Twenty-eight QCM (SEIKO EG & G, AT-cut, 9 MHz) sensors coated with different sensing films were tested and analyzed; twenty-three sensors were coated in different RTILs and five additional QCMs were coated with conventional sensing films commonly used as stationary phases in gas chromatography. The details of the sensing materials are summarized in Table 1, where the identifier used in this paper, the CAS codes, the names, and the frequency shifts caused by the deposition of the sensing layer are detailed; additional details can also be seen in Supplementary Materials Table S1.

The relative polarity of the RTIL was estimated by several factors: the length of the cation chains (shorter chains increases polarity), the cation (polarity increases in this order [Nxxxx]>[Cxmim]>[XmPY]=[Pxxx]>[Pyxx]), and the anion (polarity increases in this order: [AC]>[Cl]=[Br]=[dca]>[otf]=[BF4>[TfN2]=[PF6]) [30,31], where names correspond to abbreviations in Table 1 with x representing any number. When studying conventional films, TCP, PEG-1k, and Siponate DS10 were considered polar and OV-17 weakly polar [32,33].

The sensors were coated by dip coating carrying out the same procedure for all sensors. First, solutions of the sensing material (RTIL or conventional film) were prepared by adding around 0.1 g to 11 mL of solvent. The solutions were stirred by a magnetic agitator for 5 min. Then, the QCM sensors were submerged in the solution and a dip coater pulled out the QCM sensor. The deposition was only characterized by the QCM frequency shift that was measured with the resonant frequencies before and after the deposition (Table 1). Although no other characterization of the surface was made, the frequency shift depends on the amount of material deposited on the surface and it is a common way of characterizing sensing layers on QCMs. Additional details of the deposition are in the Supplementary Materials Table S2, including the thickness of the sensing film in nm and the solvent used.

Most of the films showed a good adhesion to the QCM sensor. Although they were coated on the surface irregularly, the stability was good. The sensing films remained on the QCM sensor surface, even if several RTILs ([C2mim][Ac], [C2mim][otf], [C2mim][dca], [C4mim][Br], [C4mim][Cl], [C4mim][Br], [C6mim][Cl], and [C8mim][Tf2N]) did not form a good coating on the surface due to dewetting effects. Other RTILs show stripes ([C2mim][otf], [C2mim][Tf2N], [C4mim][Cl] and [C6mim][Cl]), that could be related to the evaporation rate of the solvent.

Four volatile organic compounds (VOCs)—hexanol, butyl acetate, 2-hexanone, and hexanoic acid—were measured by the sensors. These VOCs were selected because they represent odorous volatile organic compounds with different functional groups (ketone, alcohol, acid, and ester) but with same number of carbons.

Because van der Waals force is one of the main factors for the interaction between gas molecules and sensing films, the partition coefficients of gases with the same number of carbons tend to be similar. In addition, according to Sauerbrey equation [34], the QCM resonant frequency shift is proportional to the mass adsorbed on its surface; volatile organic compounds with the same number of carbons typically cause a similar frequency shift. Since those molecules are difficult to distinguish, they were used in this study.

The VOC gaseous phases were generated by a continuous flow head space generation and posterior dilution. The details of the system can be found elsewhere [35], but a short summary is presented here. A mass flow controller sent 200 mL/min of bottled air thought the system. The flow was evenly split in 16 paths and the air circulated continuously through them. Eight of the paths had vials containing VOCs (5 mL), and the other 8 had empty vials. Every path could be switched from going to the sensor chamber or going to the outlet (bypassing the sensor chamber). At all times, the sensor chamber received the gases from 8 different paths (for a total of 100 mL/min). In summary, the system could mix and combine the flow going through the vials into the sensor chamber, keeping a constant flow rate through both the vials and the sensor chamber. In this experiment, two vials of each VOC were used together to increase the concentration for each gaseous VOC.

This type of system has been used before to generate odors for olfactory displays by blending pure chemical compounds [36] showing a concrete application of such device. Another application of this type of system was odor analysis and recording, where the system was used to simulate complex aromas with 5 simple components [37].

To calculate each VOC gaseous concentration generated by the odor generation system, a calibration method similar to the one used in permeation tube instrumentation was used [38]. The weight of the vials containing the odorant liquids were measured with a balance before and after the experiment. The mass flow rate per channel $\left(\phi \right)$ (0.0125 L/min) with uncertainty 5%, the time of the experiment in minutes (t) with uncertainty 1 s, and the change in mass (ΔM) in grams with uncertainty 0.002 g, were used to calculate the concentration in gr/L. Then, the general equation of ideal gases with temperature (293 K) and pressure (1 atm) and the molecular weight of each gas were used to calculate concentration in parts per million in volume (ppmv) and its uncertainty [39].

Table 2 shows the full-scale concentrations of the gaseous VOCs (with 2 channels used simultaneously). Although the gas concentrations are very different, the liquid concentration of the vials in the odor generation system was the same (100%), so the system tested liquid samples with the same concentration.

The concentrations of hexanol, butyl acetate, 2-hexanone were also measured by a photo ionization detector (PID, RAE systems, ppb RAE 3000) (64, 614, and 592 ppmv, respectively), but the concentration of hexanoic acid was too low to be measured with accuracy. The Supplementary Materials Tables S3, S4 and S5 show tests for the RTIL calibrations.

One of the main characteristics of liquid sensing films, different from rigid solid films, is that the gas absorption, in addition to the mass loading effect, also produces a change in the viscosity of the liquid film, which causes the resonant Q factor to change. The change in Q factor is directly connected to changes in the resistance of the QCM Mason equivalent circuit [40]. It has been shown that these changes can provide additional information about the gas and increase the selectivity and accuracy of electronic noses [41].

The responses of these sensors are characterized by the mass loading and the change in their viscosity, allowing a simultaneous two component measurement. The experimental setup in this work used four Vector Network Analyzers (VNA) (model: DG8SAQ VNWA v3 made by SDR-Kits company) that measured the QCMs conductance vs frequency curves [42]. This enabled the simultaneous measurement of the changes on the resonant frequency and the resistance.

The data acquisition, data analysis and visualization were all made in Matlab by software developed in the laboratory with the Statistics and Machine Learning Toolbox.

## 3. Results

### 3.1. Measurements

One set of experiments was done to characterize the sensors. In all of the experiments, the exposure to the VOC lasted for 5 min and then the sensors were left for 10 min in a flow of carrier gas (dry air) to recover. The resonant frequency and series resistance of each QCM sensor were measured every 2 s. The sensor frequency and sensor resistance curves were filtered by a five moving point median filter and then linearly interpolated to one second period. These curves were used to calculate the responses. The frequency or resistance baseline was calculated by averaging the sensor measurement during the 20 s just before the VOC exposure. The difference between the sensor frequency (or resistance) at a given time and its base line was considered to be the sensor response at that time. This base line correction was enough to compensate for the lack of sensor recovery observed after the exposition to some VOCs. With these parameters, the sensor measurement repeatability was calculated in the range of 15%; more details are in the Supplementary Materials Table S3.

**Figure 1:**
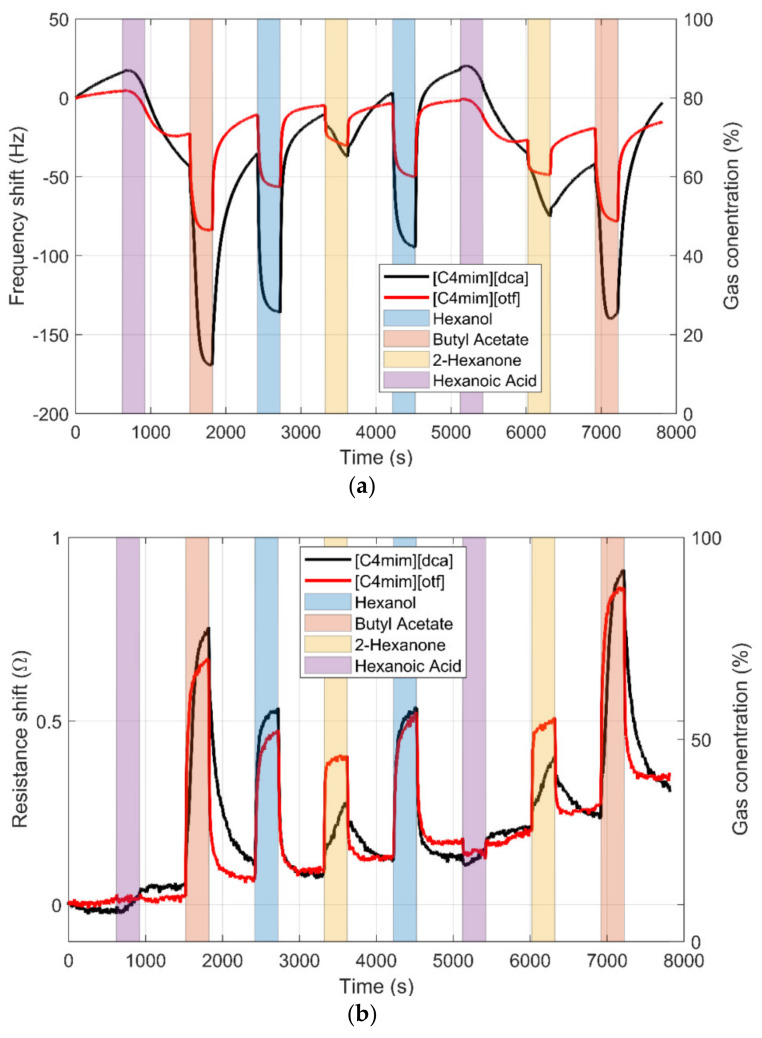
(**a**)Typical response of the sensors [C4mim][dca] and [C4mim][otf]. (**b**) Frequency response to the four VOCs. Resistance response to the four VOSs at maximum concentration.

The sensors were tested in batches of four sensors (the number of the sensor slots in the sensor chamber) within a couple of weeks after fabrication. Every sensor was exposed to each VOC 12 times at its maximum concentration. Figure 1 shows the typical responses of the sensors. The red and black lines represent the evolution of the resonant frequency (up) and series resistance (down) of two sensors (in black [C4mim][dca] and in red [C4mim][otf]. The color bars represent the intervals in which the sensors were exposed to the gases. The response is not stable; in particular, hexanoic acid has a long-lasting effect on the sensors. To compensate for this drift, the response was calculated as the increment with the baseline calculated just before the gas exposure. In addition, to capture the uncompensated variation, the response and cleaning times were kept constant and the exposures to VOCs were made in a random order. This can be seen in Figure 1, where the gases are presented eight times in a random order. In this way, the measurements capture the variation caused by the drift.

When studying all the sensors, the measurements were combined in a large dataset in which each sensor was a column and each row represented a sample.

To measure the separation power of the different sensor combinations, the Wilks lambda [43,44] was used. Wilks lambda (Λ) is given by Equation (1), where S_bg_ is the between groups variance matrix and S_wg_ is the within-group variance matrix. Wilks lambda is a measurement of the significance of the separation among the groups [43]. A lower value of Wilks lambda means a better separation. Wilks lambda is usually used for significance tests in statistical analysis, such as MANOVA, but in this case we were only interested in relative values in order to select parameters that minimize it. Because the experimental setup limits the number of sensors to four, a selection of four sensors was investigated in all cases.

(1) $$\Lambda =\left|\frac{S_{wg}}{S_{bg}+S_{wg}}\right|$$

### 3.2. RTIL Sensors

The Wilks lambda of all the possible combinations of four sensors was calculated and the lower value (better separation) was used to select the best four sensors (out of the 23 RTIL sensors). Since the data set was small enough to make this calculation within a practical amount of time, the selection was made, exploring all the possibilities instead of adding (or removing) one sensor at a time.

The frequency data were studied first: in this case, [C4mim][Cl], [Py41][Tf2N], [C4mim][otf], and [C4mim][Tf2N] sensors were selected as the best combination of sensors. The polar RTIL ([C4mim][Cl]) and non-polar RTILs ([Py41][Tf2N], [C4mim][otf], and [C4mim][Tf2N]) were selected, probably because polar RTILs have information to compensate for less polar RTILs, such as ones with [Tf2N] anion. When resistance data were studied alone, the selection was [C6mim][Br], [Py41][Tf2N], [C6mim][ Tf2N], and [C4mim][dca]. For resistance data, again, polar and non-polar anions were selected.

**Figure 2:**
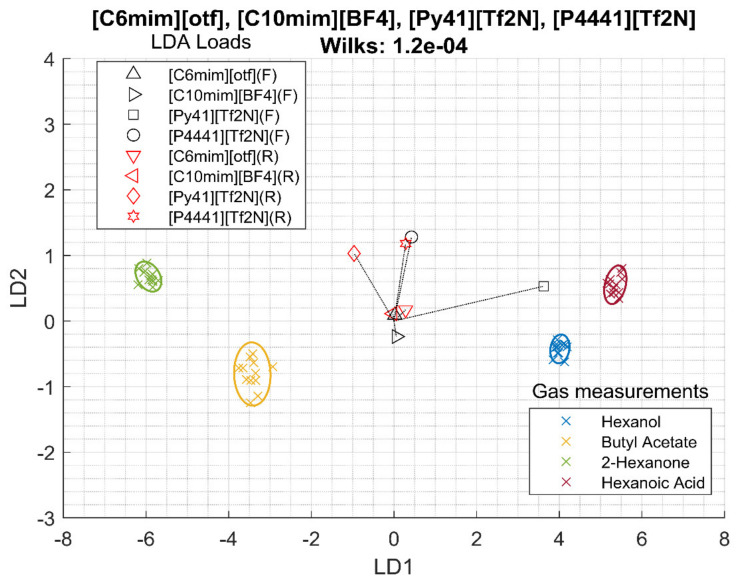
Linear discrimination analysis (LDA)of measurements of room temperature ionic liquids (RTILs) regarding the frequency (F) and resistance (R) data.

Frequency data combined with resistance data were also studied. In this case, eight features were extracted from four sensors, four frequencies and four resistances. Again, in this case, a polar RTIL ([C6mim][otf]) and three less polar RTILs ([C10mim][BF4],[P4441][Tf2N], and [Py41][Tf2N]) were selected. The linear discriminant analysis (LDA) bi-plot can be seen in Figure 2. In this plot, each cross represents one measurement and they are colored according to the VOC measured. If the separation among the groups is clear and the groups do not overlap, the system can be said to be able to separate VOCs samples, and small groups separated as much as possible is preferred. The plot also draws the LDA loads of the sensor features. A longer distance from the center means that the sensor feature is contributing more to the separation. In addition, LDA loads close to each other means that the sensor features are similar. In this case, the separation and it is quantized by the Wilks lambda as 1.2 10^−4^. Hexanol and hexanoic acid are close together, while butyl acetate and 2-hexanone are close to each other. Additionally, it can be seen that [Py41][Tf2N] has a very different weight compared to the other sensors, although [P4441][[T2fN] also has a high loading on the classification.

### 3.3. Conventional Films

**Figure 3:**
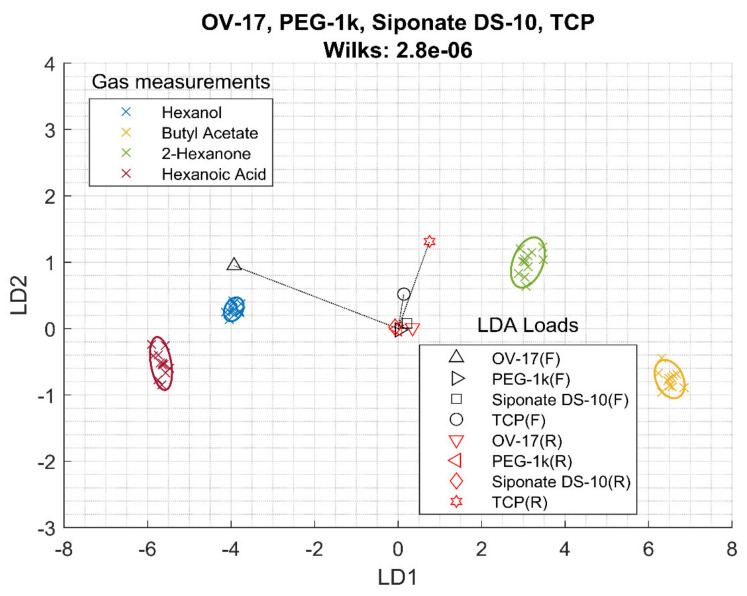
LDA of array of four best conventional films using frequency (F) and resistance (R) data.

The same analysis was made for the conventional films. For frequency, the Wilks lambda is higher than the RTIL, but this is probably related to the fact that only five conventional films were explored, instead of 23. On the other hand, the Wilks lambda, i.e., discrimination capability, for the resistance measurements is much larger than the Wilks lambda for the resistance data of the RTIL. In particular, Table S5 shows that the sensitivity of the resistance measurements to butyl acetate and 2-hexanone is very similar, making their discrimination very difficult. Finally, when the information of the frequency shift and the resistance are added together, the discrimination capability increases greatly compared with that of frequency or resistance. Figure 3 shows the LDA bi-plot for the best four sensors and the results are summarized in Table 3. Again, the classification is clear (with a lower Wilks lambda of 2.8 10^−6^) and, again, hexanol and hexanoic acid are close together as well as butyl acetate and 2-hexanone. In this case, OV-17 and TCP contribute more to the LDA classification.

### 3.4. All Sensors Together

Finally, the combination of the RTIL with conventional films was studied. After adding all sensors together, the same analysis of Wilks lambda was made.

**Figure 4:**
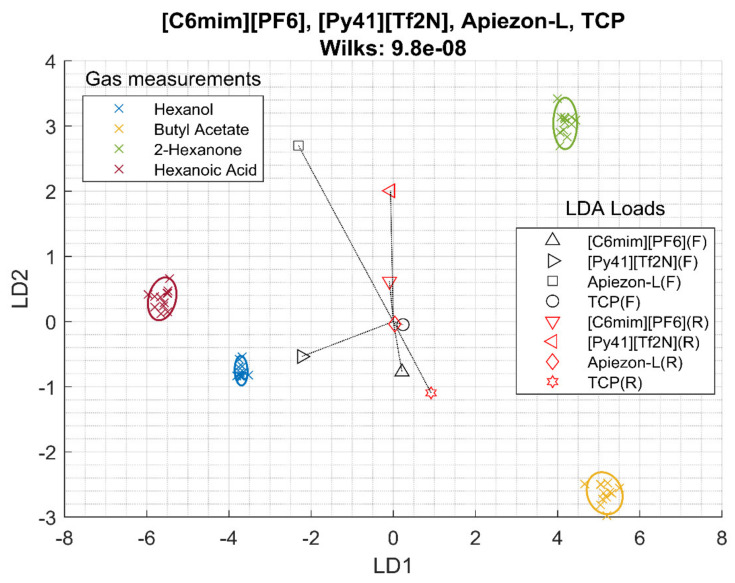
LDA of all sensors and all data points using frequency (F) and resistance (R) data.

When using frequency from four sensors, the selection is [Py41][Tf2N], [C4mim][Cl], [C4mim][otf] and OV-17. The Wilks lambda was 1.3 10^−6^, much lower than both the conventional films and the RTIL sensors. In resistance, the selection of sensors changes and becomes [Py41][Tf2N], [C6mim][Br], [C6mim][PF6] and TCP, and its Wilks lambda is 6.8 10^−5^. When combining frequency and resistance, the sensor selection is [Py41][Tf2N], [C6mim][PF6], Apiezon-L, and TCP, and it gives a Wilks lambda of 9.8 10^−8^. Figure 4 shows the LDA bi-plot of the frequency and resistance measurements. In this case, only hexanol and hexanoic acid remain close together, as butyl acetate and 2-hexanone get separated; this is reflected in the lower Wilks Lambda. In this case, more sensor features contribute to the separation and RTIL, and conventional films show different loadings.

In all cases, [Py41][Tf2N] was selected. The combination of sensors includes OV-17 (which is weakly polar) or TCP, which is polar, so again polar and non-polar materials are selected. In addition, one RTIL and one conventional film are always selected as part of the sensor array. Table 3 summarizes all the results.

The less commonly chosen conventional film is Siponate DS-10, despite being selected when inspecting only conventional films. This is probably because it is an organic salt, similarly to RTILs, and its information or selectivity is related to them.

### 3.5. Validation of Sensor Array with Two Aromas

**Figure 5:**
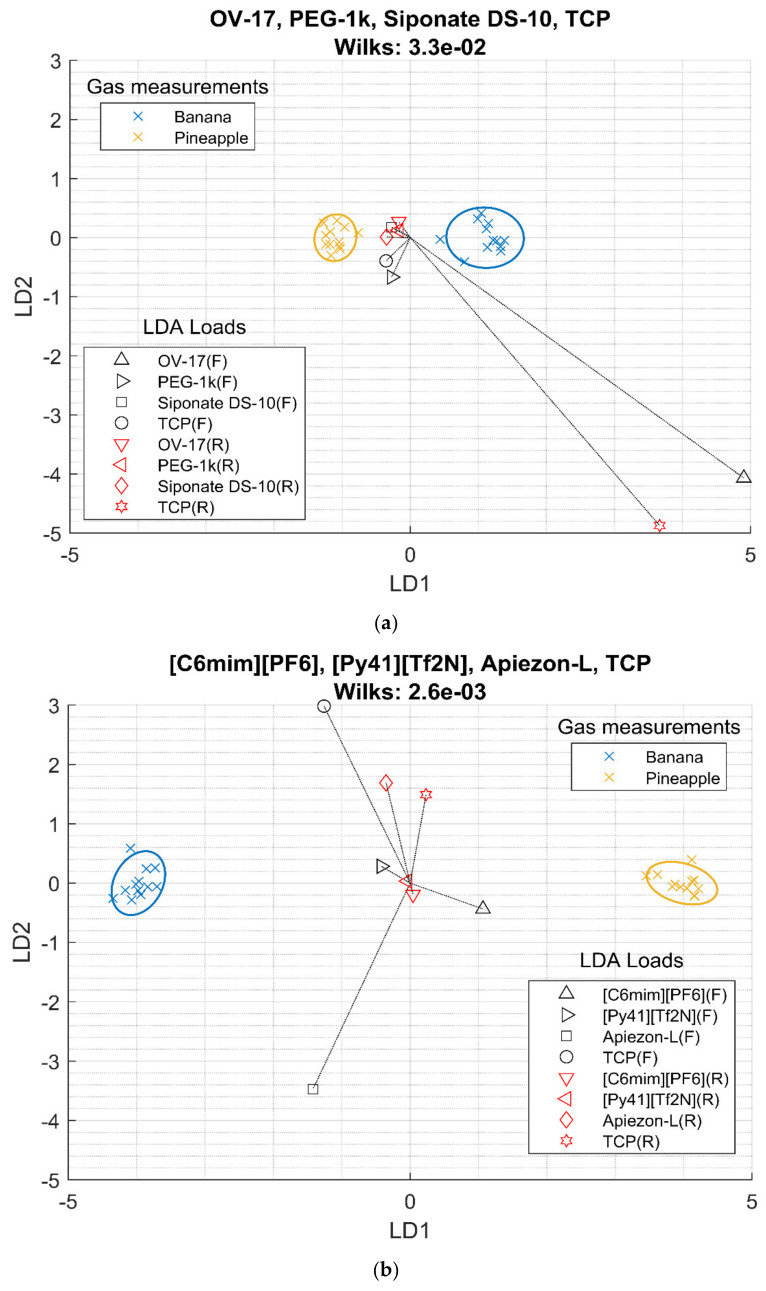
LDA bi-plot for the best sensor array using frequency (F) and resistance (R) data of the 2 scents measured. (**a**) Conventional films. (**b**) Best sensing film selection.

To validate the study, the best selection of a sensor to build an array measuring frequency and resistance was used to classify two different flavors: banana and pineapple. The aromas were flavors provided by Hassewaga Corporation, and they were similar as they evoked mature fruits. The organoleptic similarity between the two samples is higher than those among the previous four pure VOCs samples that evoked different aromatics. The same test was made with the best four conventional films to compare results. Figure 5 shows the LDA bi-plot of both experiments.

The sensors showed a similar response pattern of the pure VOCs; for conventional films only, OV-17 and TCP had a higher response. In the case of RTIL combined with conventional films, [C6mim][PF6] had a higher contribution compared with [Py41][Tf2N]. The patterns of these two flavors were similar, only using the conventional films (with a Wilks lambda of 3.3 10^−2^). On the other hand, the separation using the selected sensors (from RTIL + conventional films) was larger than those only using conventional films (with a Wilks lambda of 2.6 10^−3^), getting better separation again even if the selection of sensors was optimized for the four VOCs samples.

## 4. Discussion

Looking at Figure 2, Figure 3 and Figure 4 and the Wilks Λ, it can be concluded that the addition of resistance data improves the array discrimination power of the VOCs. In Table 3, it can be seen that, for each column, the Wilks lambda for frequency together with resistance is lower than frequency alone, and frequency alone is better than resistance.

To test whether frequency combined with resistance data is better than only frequency, the eight sensors that gave the lowest Wilks Λ were selected, taking only frequency data. The Wilks Λ of this set of eight sensors is 5.6 10^−6^ and 1.72 10^−7^ for RTILs and all sensors, respectively (Table 3), slightly higher values than the Wilks Λ calculated with the frequency and resistance data of the four best sensors (4.6 10^−6^ and 9.8 10^−8^). This shows that the addition of the four resistance features is slightly better than adding four more frequency-only sensors.

Several chemical properties of RTILs make them excellent for gas sensor sensing materials; chemical stability, which lower drift and increases stability; low volatility, which also increases sensor stability; variety of sensing films’ characteristics that improves sensor specificity as proved in this paper.

However, some other problems need further investigation; the adhesion to the surfaces is not always good, and their liquid nature could impose limits on the coating stability and could cause long-term drift that has not been studied in this work. These adhesion and coating stability problems are alleviated by the small thinness of the sensing film.

Other possible problem that we have not studied is the temperature and humidity influence. They can limit the stability of the measurements. Although the temperature affects the QCM resonator characteristics, such as the resonant frequency [45], the resonant frequency of the AT-cut QCM sensors is stable at a temperature of around 25 °C [46], so the temperature influence will be caused mainly by the sensing film properties, such as the partition coefficient [47] and its viscoelastic coefficients. The humidity affects the properties of the sensing film and can act as an interfering compound [48]. In this work, we kept these parameters constant, so this limits the practical applications to uses such as odor recording [37].

Additional studies can be done to investigate the sensor fabrication reproducibility. While it was not studied in this paper, many scientific studies have remarked on the high reproducibility of the dip coating technique [49]. Other studies that have investigated the sensor reproducibility of QCM sensors coated with conventional films with other methods have shown a reproducibility on the partition coefficient between 5% and 30% [50], bounding the reproducibility of the film coatings. The frequency shift caused by the deposition was used to monitor and characterize the sensor fabrication. While this accurately monitors the amount of material deposited, different sensors will have different responses and a calibration of each sensor array will be needed. Sensor reproducibility is a common problem of the electronic noses, making calibrations for each particular array necessary.

## 5. Concussions

In this paper, 23 RTIL films were studied as sensing films on QCM gas VOCs sensors to evaluate their performance as sensing elements on an electronic nose. They were also compared to five gas chromatography stationary phases to determine whether they could improve arrays made by conventional films.

Two key points can be obtained from this analysis. First, frequency data are better for classifying than resistance data, improving the Wilks lambda by a factor of 3.4 for RTILs, but the addition of resistance data to frequency data improves the array separation of the VOCs, improving the Wilks lambda by a factor of 7.8 for RTILs.

Second, the addition of conventional films to RTIL films further improves the discrimination power compared to only RTIL films or only conventional films by a factor between 46.9 (compared to RTIL using frequency and resistance data) and 93.9 (compared to conventional films using frequency and resistance data). The best sensor combinations include at least one RTIL and one conventional film. This indicates that both kinds of sensor films have complementary information that improves the classification.

These two conclusions open the door to the improvement of electronic noses with the incorporation of these RTILs as new sensor materials and the interrogation of two different physical parameters on QCM gas sensors.

## Figures and Tables

**Table 1:** Details of the sensing materials used in the study and the quartz crystal microbalance (QCM) frequency shift of the sensing layer deposition.

Sensing Material			Deposition Frequency Shift
Identifier	CAS #	Full Name	(Hz)
[C2mim][otf]	145022-44-2	1-Ethyl-3-methylimidazolium triflate	1688
[C2mim][Tf2N]	174899-82-2	1-Ethyl-3-methylimidazolium	1184
		bis(trifluoromethylsulphonyl)imide	
[C2mim][Ac]	143314-17-4	1-Ethyl-3-methylimidazolium acetate	1584
[C2mim][dca]	370865-89-7	1-Ethyl-3-methylimidazolium dicyanamide	1514
[C4mim][dca]	448245-52-1	1-butyl-3-methylimidazolium dicyanamide	1300
[C4mim][otf]	174899-66-2	1-Butyl-3-methylimidazolium triflate	1149
[C4mim][Ac]	284049-75-8	1-Butyl-3-methylimidazolium acetate	967
[C4mim][Cl]	79917-90-1	1-butyl-3-methylimidazolium chloride	1006
[C4mim][Tf2N]	174899-83-3	1-butyl-3-methylimidazolium	1509
		bis(trifluoromethylsulfonyl)imide	
[C4mim][Br]	85100-77-2	1-butyl-3-methylimidazolium bromide	1200
[C6mim][Tf2N]	382150-50-7	1-hexyl-3-methylimidazolium	1194
		bis[(trifluoromethyl)sulfonyl]imide	
[C6mim][otf]	460345-16-8	1-hexyl-3-methylimidazolium	1268
		trifluoromethanesulfonate	
[C6mim][PF6]	304680-35-1	1-hexyl-3-methylimidazolium hexafluorophosphate	1042
[C6mim][Br]	85100-78-3	3-hexyl-1-methyl-1H-imidazolium bromide	1119
[C6mim][Cl]	171058-17-6	1-hexyl-3-methylimidazolium chloride	1300
[C8mim][PF6]	304680-36-2	1-octyl-3-methylimidazolium	1438
		Hexafluorophosphate	
[C8mim][Tf2N]	178631-04-4	1-octyl-3-methylimidazolium	1175
		bis[(trifluoromethyl)sulfonyl]imide	
[C10mim][BF4]	244193-56-4	1-Decyl-3-methylimidazolium	1055
		tetrafluoroborate	
[BmPY][Tf2N]	623580-02-9	1-Butyl-1-methylpiperidinium	1182
		bis(trifluoromethylsulphonyl)imide	
[N4111][Tf2N]	324575-10-2	Butyltrimethylammonium	1561
		bis(trifluoromethanesulfonyl)imide	
[Py41][Tf2N]	223437-11-4	1-Butyl-1-methylpyrrolidinium bis(trifluoromethanesulfonyl)imide	1163
[P4441][Tf2N]	324575-10-2	Tributylmethylphosphonium	1293
		bis(trifluoromethanesulfonyl)imide	
[N1888][Tf2N]	258273-75-5	Methyltrioctylammonium	1156
		bis(trifluoromethylsulphonyl)imide	
TCP	1330-78-5	Tricresyl phosphate	1352
APIEZON-L	8009-03-08	Petrolatum (Vaseline)	1339
PEG 1k	25322-68-3	Poly(ethylene glycol) 1,000	1169
OV-17	63148-58-3	Methyl Phenyl Silicone Oil	1179
SIPONATE DS-10	25155-30-0	Sodium dodecylbenzenesulfonate	1230

**Table 2:** Gaseous volatile organic compound (VOC) concentrations at 100% concentration and the uncertainty associated.

Gas	Hexanol	Butyl Acetate	2-Hexanone	Hexanoic Acid
Concentration (ppmv)	62.9	758.7	753.8	9.0
Uncertainty (%)	25.0	5.3	5.2	148.7

**Table 3:** Wilks lambda and the best sensor selection of different number of sensor taken from different sets (first column: RTIL, second column: conventional films, and third column: all sensors). (F) refers to frequency data and (R) refers to resistance data.

	RTIL		Conventional Films		All Sensors	
	Sensors	Wilks Λ	Sensors	Wilks Λ	Sensors	Wilks Λ
Frequency Data (4 features)	[Py41][Tf2N](F)	3.5 × 10^−5^	OV-17(F)	1.6 × 10^−4^	[Py41][Tf2N](F)	1.3 × 10^−6^
	[C4mim][Cl](F)		Siponate DS-10(F)		[C4mim][Cl](F)	
	[C4mim][otf](F)		TCP(F)		[C4mim][otf](F)	
	[C4mim][Tf2N](F)		Apiezon-L(F)		OV-17(F)	
Resistance Data (4 features)	[Py41][Tf2N](R)	1.2 × 10^−4^	OV-17(R)	4.2 × 10^−3^	[Py41][Tf2N](R)	6.8 × 10^−5^
	[C4mim][dca](R)		Siponate DS-10(R)		[C6mim][Br](R)	
	[C6mim][Br](R)		TCP(R)		[C6mim][Tf2N](R)	
	[C6mim][Tf2N](R)		PEG-1k(R)		TCP(R)	
Frequency and Resistance Data (8 features)	[Py41][Tf2N](F&R)	4.6 × 10^−6^	OV-17(F&R)	9.2 × 10^−6^	[Py41][Tf2N](F&R)	9.8 × 10^−8^
	[P4441][Tf2N](F&R)		Siponate DS-10(F&R)		[C6mim][PF6](F&R)	
	[C6mim][otf](F&R)		TCP(F&R)		TCP(F&R)	
	[C10mim][BF4](F&R)		PEG-1k(F&R)		Apiezon-L(F&R)	
Frequency (8 features)	[C4mim][otf](F)	5.6 × 10^−6^			[Py41][Tf2N](F)	1.7× 10^−7^
	[Py41][Tf2N](F)				OV-17(F)	
	[C4mim][Cl](F)				[C10mim][BF4](F)	
	[C4mim][Tf2N](F)				[C4mim][otf](F)	
	[C6mim][Br](F)				Apiezon-L(F)	
	[C4mim][Br](F)				[C4mim][Cl](F)	
	[C6mim][Tf2N](F)				[C4mim][Tf2N](F)	
	[N4111][Tf2N](F)				[C6mim][otf](F)	

## Supplementary Materials

The following are available online at https://www.mdpi.com/1424-8220/20/14/4026/s1.
