# Supplementary material for: Study of Room Temperature Ionic Liquids as Gas Sensing Materials in Quartz Crystal Microbalances
Source: Sensors (Basel). 2020 Jul 20;20(14):4026. doi: 10.3390/s20144026 (PMC7411987; doi:10.3390/s20144026)
Supplement: Supplementary file 1 [file sensors-20-04026-s001.pdf]

sensors

Supplementary

Study of Room Temperature Ionic Liquids as Gas Sensing Materials in Quartz Crystal Microbalances.

Manuel Aleixandre and Takamichi Nakamoto *

Institute of Innovative Research, Tokyo Institute of Technology, Yokohama, 226-8503, Japan; nakamoto.t.ab@m.titech.ac.jp
* Correspondence: nakamoto.t.ab@m.titech.ac.jp

A1. RTILs used in the study

Table S1. Details of the sensing materials used in the study. All data at 293 K and 101.325 Pa except (a) at 298, (b) 373 and (c) 303.15 K.

Sensing material			Sensor thickness			
Identifier	CAS #	Full name	Density (kg/m^3)	Ref.	Viscosity (Pa s)	Ref.
[C2mim][otf]	145022-44-2	1-Ethyl-3-methylimidazolium triflate	1386	[1]	0.04	[2]
[C2mim][Tf2N]	174899-82-2	1-Ethyl-3-methylimidazolium bis(trifluoromethylsulphonyl)imide	1526	[3]	0.03	[4]
[C2mim][Ac]	143314-17-4	1-Ethyl-3-methylimidazolium acetate	1103	[5]	0.20	[1]
[C2mim][dca]	370865-89-7	1-Ethyl-3-methylimidazolium dicyanamide	1107	[6]	0.02	[6]
[C4mim][dca]	448245-52-1	1-butyl-3-methylimidazolium dicyanamide	1064	[7]	0.04	[8]
[C4mim][otf]	174899-66-2	1-Butyl-3-methylimidazolium triflate	1306	[9]	0.10	[10]
[C4mim][Ac]	284049-75-8	1-Butyl-3-methylimidazolium acetate	1055	[11]	0.55	[12]
[C4mim][Cl]	79917-90-1	1-butyl-3-methylimidazolium chloride	1074 (a)	[13]	41.00	[14]
[C4mim][Tf2N]	174899-83-3	1-butyl-3-methylimidazolium bis(trifluoromethylsulfonyl)imide	1442	[7]	0.06	[15]
[C4mim][Br]	85100-77-2	1-butyl-3-methylimidazolium bromide	1300 (a)	[16]	0.06 (b)	[17]
[C6mim][Tf2N]	382150-50-7	1-hexyl-3-methylimidazolium bis[(trifluoromethyl)sulfonyl]imide	1371	[18]	0.09	[18]
[C6mim][otf]	460345-16-8	1-hexyl-3-methylimidazolium trifluoromethanesulfonate	1235	[19]	0.12 (c)	[10]
[C6mim][PF6]	304680-35-1	1-hexyl-3-methylimidazolium hexafluorophosphate	1295	[20]	0.587	[20]
[C6mim][Br]	85100-78-3	3-hexyl-1-methyl-1H-imidazolium bromide	1228	[21]	6.94	[21]
[C6mim][Cl]	171058-17-6	1-hexyl-3-methylimidazolium chloride	1034	[22]	18.00	[14]
[C8mim][PF6]	304680-36-2	1-octyl-3-methylimidazolium Hexafluorophosphate	1235	[23]	0.98	[24]
[C8mim][Tf2N]	178631-04-4	1-octyl-3-methylimidazolium bis[(trifluoromethyl)sulfonyl]imide	1328	[25]	0.12	[4]

[C10mim][BF4]	244193-56-4	1-Decyl-3-methylimidazolium tetrafluoroborate	1072	[25]	0.93	[14]
[BmPY][Tf2N]	623580-02-9	1-Butyl-1-methylpiperidinium bis(trifluoromethylsulphonyl)imide	1387	[26]	0.26	[26]
[N4111][Tf2N]	324575-10-2	Butyltrimethylammonium bis(trifluoromethanesulfonyl)imide	1397	[27]	0.14	[27]
[Py41][Tf2N]	223437-11-4	1-Butyl-1-methylpyrrolidinium bis(trifluoromethanesulfonyl)imide	1400	[5]	0.10	[28]
[P4441][Tf2N]	324575-10-2	Tributylmethylphosphonium bis(trifluoromethanesulfonyl)imide	1258	[29]	0.30	[29]
[N1888][Tf2N]	258273-75-5	Methyltrioctylammonium bis(trifluoromethylsulphonyl)imide	1105	[30]	0.68	[30]
TCP	1330-78-5	Tricresyl phosphate	1160	[31]	0.72	[31]
APIEZON-L	8009-03-08	Petrolatum (Vaseline)	896	[32]	64.00	[32]
PEG 1k	25322-68-3	Poly(ethylene glycol) 1,000	1200	[33]	Solid	[34]
OV-17	63148-58-3	Methyl Phenyl Silicone Oil	1092	[35]	0.10	[36]
SIPONATE DS-10	25155-30-0	Sodium dodecylbenzenesulfonate	1000	[37]	Solid	

Table S2 detail the deposition parameters used for each sensor. The materials used in the study were bought from the following companies:

1. Solvents: acetone (99.5%) from Junsei, chloroform (99% with ethanol as stabilizer 0.3% to 1%) and ethanol (99.5%) from wako, methanol (99.8%) from TCI.
2. The VOCs used (hexanol, 2-hexanone, hexanoic acid, and buty acetate) were al bough from TCI.
3. Ionic liquids: all bought from TCI except [C4mim][ac] bought from Sigma Aldrich and [BmPY][T2fN] that was bought from IOLITEC.
4. The conventional films were bought from Shimazdu Corporation except TCP that was bought from TCI.

Table 2. Details of the sensors used in the study.

Coating Solution				Deposition thickness		
Sensing material	Solvent name	Concentration	Deep Coating pull-up Speed	ΔFs	ΔM	Thickness
		(μg/mL)	(μm/s)	(Hz)	kg	(nm)
[C2mim][otf]	Ethanol	9.59	100	1688	1.39E-09	66.64
[C2mim][T2fN]	Ethanol	9.87	100	1184	1.23E-09	48.11
[C2mim][Ac]	Ethanol	6.71	200	1584	1.04E-09	49.98
[C2mim][dca]	Ethanol	9.38	100	1514	1.08E-09	51.07
[C4mim][dca]	Acetone	12.95	70	1300	1.38E-09	56.04
[C4mim][otf]	Acetone	12.52	50	1149	1.26E-09	48.24
[C4mim][Ac]	Acetone	8.42	500	967	1.24E-09	57.01
[C4mim][Cl]	Chloroform	5.93	200	1006	1.26E-09	46.45
[C4mim][Tf2N]	Acetone	9.74	500	1509	1.62E-09	57.07
[C4mim][Br]	Chloroform	9.43	600	1200	1.28E-09	47.31
[C6mim][Tf2N]	Acetone	9.39	1500	1194	1.67E-09	60.93
[C6mim][otf]	Acetone	10.93	500	1268	1.25E-09	45.32
[C6mim][PF6]	Acetone	9.45	100	1042	1.7E-09	78.71
[C6mim][Br]	Chloroform	9.41	800	1119	1.62E-09	74.21
[C6mim][Cl]	Chloroform	10.40	600	1300	1.81E-09	66.20
[C8mim][PF6]	Acetone	9.35	100	1438	1.27E-09	42.33
[C8mim][Tf2N]	Acetone	9.50	100	1175	1.2E-09	49.65
[C10mim][BF4]	Methanol	9.40	150	1055	1.39E-09	68.58
[BmPY][Tf2N]	Acetone	9.65	100	1182	1.28E-09	50.25
[N4111][Tf2N]	Acetone	9.89	600	1561	1.36E-09	55.98
[Py41][Tf2N]	Acetone	9.55	600	1163	1.45E-09	61.70
[P4441][Tf2N]	Acetone	9.26	100	1293	1.44E-09	81.61
[N1888][Tf2N]	Acetone	9.79	150	1156	1.25E-09	53.14
TCP	Ethanol	10.21	100	1352	1.26E-09	58.90
APIEZON-L	Chloroform	10.55	1600	1339	1.32E-09	67.09
PEG 1k	Chloroform	10.31	1500	1169	1.12E-09	43.62
OV-17	Chloroform	10.06	1500	1179	1.54E-09	63.54
SIPONATE DS-10	Chloroform	8.77	1500	1230	1.13E-09	53.70

A2. Calibration

A2.1. Measurements

A series of calibration experiments were used to analyze the sensitivity of every sensor to the four gases. In these measurements, each sensor was exposed to the four different gases at concentrations of 100%, 50%, 25%, and 10% of the maximum concentration. With these data, the slope, i.e., sensor's sensitivity to that gas, was calculated using a linear least squares method. For some sensors, more measurements were made in order of test the repeatability. Figures S1 to S4 show these measurements. The repeatability was calculated as the root mean squares and it is represented in Table S3. The sensitivities for all gases in frequency shift and resistance shift are in Tables S4 and S5, respectively.

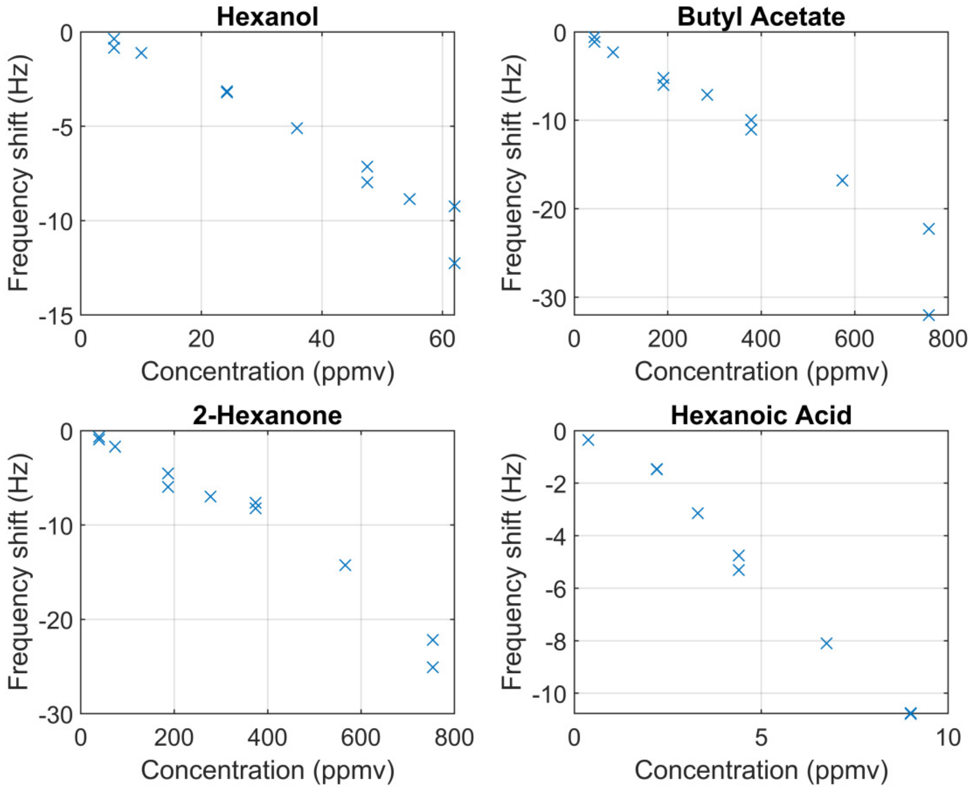

Figure S2. Repeatability measurement for [BmPY][Tf2N]

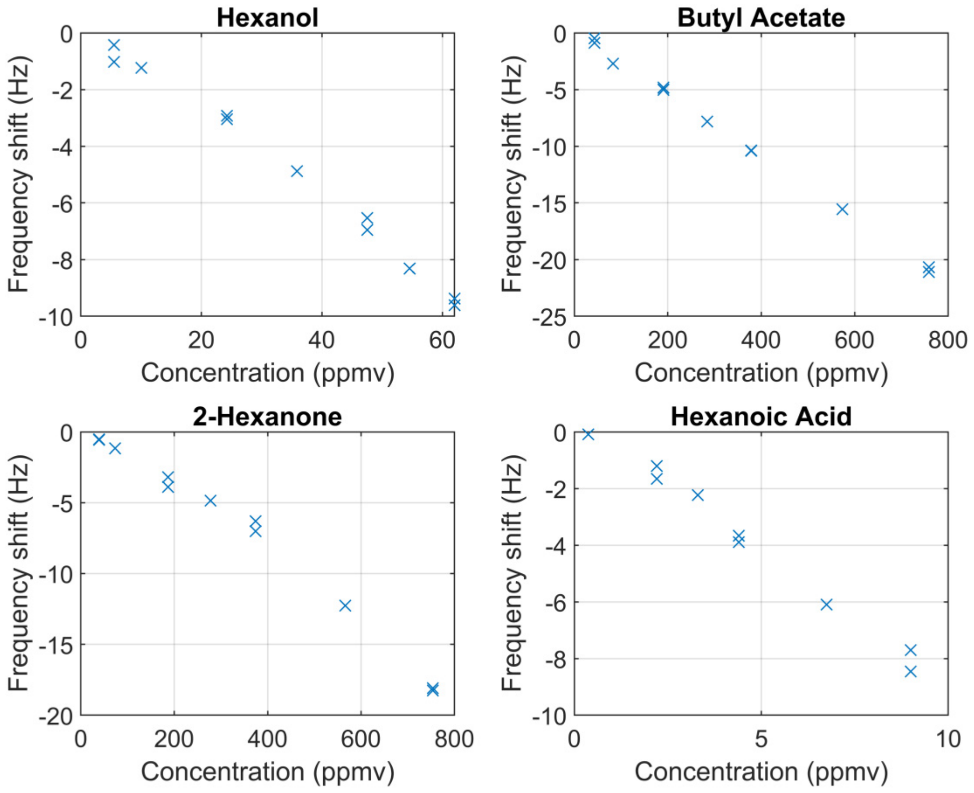

Figure S3. Repeatability measurement for [N1888][Tf2N]

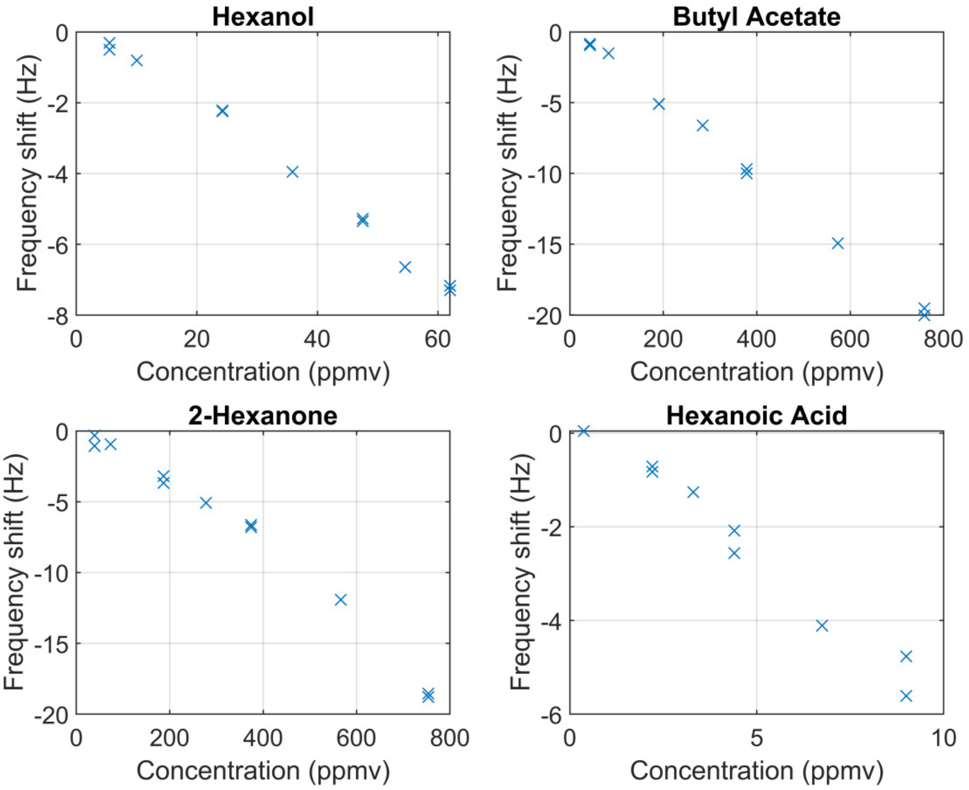

Figure S4. Repeatability measurement for [C8mim][Tf2N]

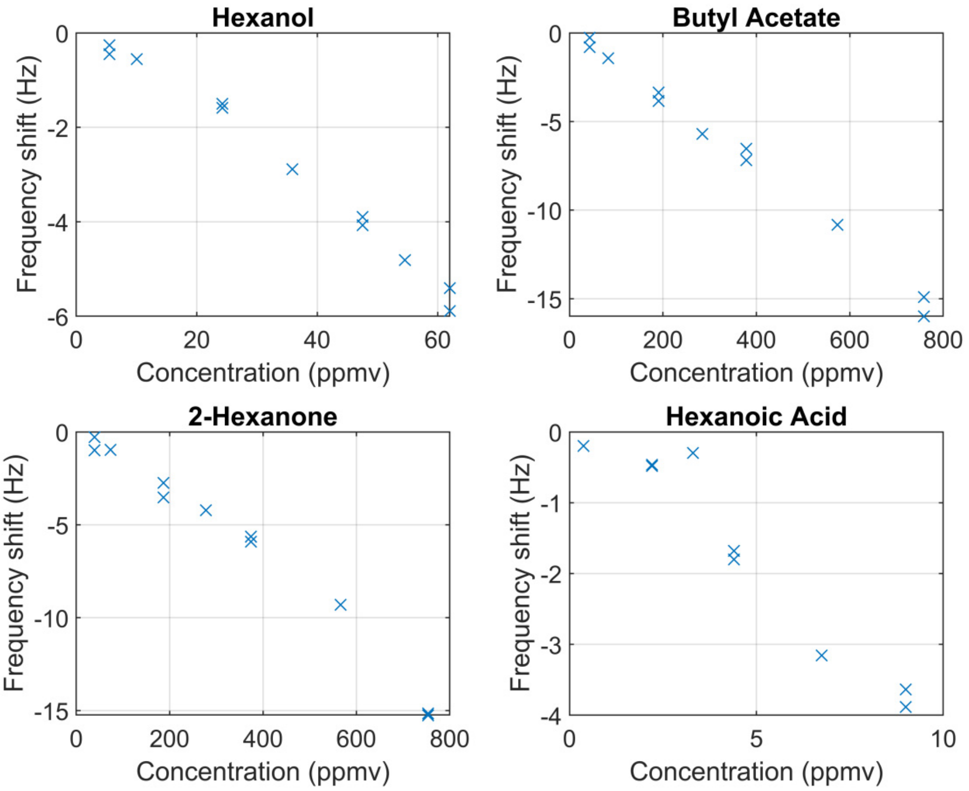

Figure S5. Repeatability measurement for [P4441][Tf2N]

Table S3 shows the repeatability calculated as the root mean square of the differences on frequency response for the same concentration, then divided by the max frequency to obtain the relative root mean square (RRMS). The maximum error was 15% and the average was 5.3 %

Table S3. Measurement repeatability in RRMS (%) of frequency shift for sensors [P4441][Tf2N], [C8mim][Tf2N], [N1888][Tf2N], and [BmPY][Tf2N].

	Hexanol	Butyl Acetate	2-Hexanone	Hexanoic Acid
[P4441][Tf2N]	4.71	1.67	4.04	12.90
[C8mim][Tf2N]	4.54	1.48	1.45	15.39
[N1888][Tf2N]	3.59	2.50	2.74	6.56
[BmPY][Tf2N]	4.11	10.08	6.21	2.97

Table S4 shows all the sensitivities in frequency shift and their R^2 for the linear adjustments. The sensitivity was transformed from Hz/% to Hz/ppmv by dividing by the max concentrations represented in Table 3 and multiplied by 100. Low R^2 could mean high deviation from linearity or poor measurement repetitivity (caused perhaps by slow sensor recovery or sensor drift). Higher sensitivities and high R^2 are desired, as they would increase the precision of the measurements.

Table S4. Sensor sensitivities to the four gases in frequency. The sensitivity to the different gases are marked in bars. Low R^2 are marked in red.

Type	Name	Hexanol		Butyl Acetate		2-Hexanone		Hexanoic Acid	
		Sensitivity (Hz/ppmv)	R^2	Sensitivity (Hz/ppmv)	R^2	Sensitivity (Hz/ppmv)	R^2	Sensitivity (Hz/ppmv)	R^2
RTIL	[C2mim][otf]	-0.1489	0.950	-1.2927	0.930	-1.4606	0.983	-0.0395	0.987
	[C2mim][Tf2N]	-0.0071	0.926	-0.1086	0.972	-0.0791	0.970	-0.0004	0.899
	[C2mim][Ac]	-0.7010	0.755	-10.2167	0.777	-3.7312	0.932	-0.0195	0.970
	[C2mim][dca]	-0.0286	0.995	-0.3317	0.986	-0.2260	0.972	-0.0023	0.880
	[C4mim][dca]	-0.0321	0.999	-0.9557	0.998	-0.9752	0.976	-0.0031	0.998
	[C4mim][otf]	-0.0273	0.997	-0.8313	0.999	-0.8058	0.979	-0.0019	0.997
	[C4mim][Ac]	-0.0241	0.996	-0.8711	1.000	-0.9113	0.981	-0.0015	0.984
	[C4mim][Cl]	-0.0309	0.984	-1.0873	1.000	-1.1356	0.983	-0.0024	0.992
	[C4mim][Tf2N]	-0.1804	0.996	-2.0745	0.944	-1.3524	0.998	-0.0136	0.968
	[C4mim][Br]	-0.3101	0.999	-1.1668	0.819	-1.9565	0.999	-0.0570	0.989
	[C6mim][Tf2N]	-0.1961	0.976	-2.4044	0.967	-1.9088	0.996	-0.0279	0.988
	[C6mim][otf]	-0.1014	0.681	-1.5802	0.986	-1.1638	0.958	0.0446	0.955
	[C6mim][Br]	-0.3609	0.897	-2.2214	0.912	-2.3198	1.000	-0.0134	0.964
	[C6mim][Cl]	-0.1550	0.963	-0.6525	0.881	-0.9212	1.000	-0.0181	0.992
	[C6mim][PF6]	-0.0192	0.956	-0.4743	0.998	-0.4694	0.985	-0.0021	0.993
	[C8mim][PF6]	-0.0436	0.988	-0.7331	0.990	-0.8275	0.987	-0.0070	0.979
	[C8mim][Tf2N]	-0.0274	0.994	-0.5796	0.998	-0.5414	0.978	-0.0025	0.975
	[C10mim][BF4]	-0.1043	0.966	-0.7098	0.994	-0.7659	0.997	-0.0133	0.845
	[BmPY][Tf2N]	-0.0396	0.960	-0.7801	0.929	-0.6660	0.958	-0.0050	0.989
	[N4111][Tf2N]	-0.1011	0.950	-1.3395	0.870	-0.7758	0.999	-0.0201	0.989
	[Py41][Tf2N]	-0.4551	0.916	-2.5746	0.929	-1.7941	0.958	-0.1068	0.990
	[P4441][Tf2N]	-0.0210	0.986	-0.4479	0.993	-0.4330	0.976	-0.0018	0.936
	[N1888][Tf2N]	-0.0346	0.990	-0.6096	0.998	-0.5312	0.981	-0.0037	0.990
Conventional films	TCP	-0.0094	0.870	-0.2335	0.982	-0.1059	0.954	-0.0007	0.992
	Apiezon-L	-0.0067	0.951	-0.3538	0.943	-0.1836	0.984	0.0003	0.976
	PEG-1k	-0.0353	0.992	-0.3721	0.907	-0.2331	0.986	-0.0015	0.971
	OV-17	-0.0080	0.970	-0.1911	0.761	-0.0891	0.907	-0.0003	0.778
	Siponate DS-10	-0.0117	0.693	0.3250	0.981	0.2273	0.957	-0.0019	0.955

In general, the sensitivity of the RTIL is higher than that of the conventional films, indicating they are a good choice for gas sensors. From these measurements, [C2mim][Ac], [Py41][Tf2N], [C4mim][Br], [C6mim][Br], [C6mim][Tf2N], and [C2mim][otf] seem appropriate sensors, as their sensitivity and R^2 are both high. [C2mim][Ac] particularly shows a very high response, even if the R^2 is not very good. Some sensors have a small response: [C2mim][Tf2N] and [C2mim][dca]. Other sensors have a different behavior depending on the gases; [C4mim][dca], [C4mim][otf], [C4mim][Ac], [C4mim][Cl], [C8mim][Tf2N], and [P4441][Tf2N] respond well to 2-hexanone and butyl acetate but little to hexanoic acid and hexanol; the opposite happens to [C4mim][Tf2N], [Py41][Tf2N] and the conventional films. No conclusion could be drawn about the effect of the anions size or the cations size on the sensitivity.

The same analysis was made for the resistance measurements (Table S5). Sensors that have high overall resistance sensitivities and high R^2 (good linearity of the calibration and low noise) are: [BmPY][Tf2N], [N1888][Tf2N], [C8mim][Tf2N], [P4441][Tf2N], for RTILs, and TCP in conventional films. [C2mim][Tf2N], [C4mim][Ac], [C4mim][Br] have small responses.

Table S5. Sensor sensitivities to the four gases in resistance. The sensitivity to the different gases are marked in bars. Low R^2 are marked in red.

Type		Hexanol		Butyl Acetate		2-Hexanone		Hexanoic Acid	
		Sensitivity (Ω/ppmv)	R^2	Sensitivity (Ω/ppmv)	R^2	Sensitivity (Ω/ppmv)	R^2	Sensitivity (Ω/ppmv)	R^2
RTIL	[C2mim][otf]	0.0005	0.617	0.0132	0.891	0.0131	0.973	0.0001	0.881
	[C2mim][Tf2N]	-0.0001	0.204	0.0004	0.101	0.0005	0.171	0.0000	0.040
	[C2mim][Ac]	-0.0333	0.230	0.1881	0.463	0.0249	0.943	0.0001	0.819
	[C2mim][dca]	0.0011	0.938	0.0187	0.968	0.0119	0.971	0.0001	0.946
	[C4mim][dca]	0.0003	0.781	0.0080	0.999	0.0096	0.965	0.0000	0.792
	[C4mim][otf]	0.0002	0.902	0.0051	0.999	0.0063	0.998	0.0000	0.829
	[C4mim][Ac]	0.0000	0.009	0.0027	0.933	0.0039	0.874	0.0000	0.731
	[C4mim][Cl]	0.0003	0.997	0.0133	0.986	0.0174	0.982	0.0000	0.346
	[C4mim][Tf2N]	0.0002	0.754	0.0050	0.949	0.0030	0.821	0.0000	0.155
	[C4mim][Br]	0.0049	0.727	0.0004	0.000	0.0066	0.309	0.0007	0.319
	[C6mim][Tf2N]	0.0010	0.893	0.0148	0.997	0.0124	0.970	0.0001	0.959
	[C6mim][otf]	0.0194	0.623	0.0185	0.031	0.0275	0.663	0.0123	0.910
	[C6mim][Br]	0.0147	0.876	0.0970	0.988	0.0536	0.984	0.0001	0.980
	[C6mim][Cl]	0.0013	0.976	0.0127	0.814	0.0110	0.986	0.0002	0.988
	[C6mim][PF6]	0.0001	0.514	0.0048	0.901	0.0061	0.979	0.0000	0.843
	[C8mim][PF6]	0.0008	0.959	0.0189	0.985	0.0228	0.970	0.0001	0.985
	[C8mim][Tf2N]	-0.0274	0.994	-0.5796	0.998	-0.5414	0.978	-0.0025	0.975
	[C10mim][BF4]	0.0050	0.994	0.0179	0.066	0.0302	0.918	0.0020	0.936
	[BmPY][Tf2N]	-0.0396	0.960	-0.7801	0.929	-0.6660	0.958	-0.0050	0.989
	[N4111][Tf2N]	-0.0047	0.307	-0.0016	0.002	0.0085	0.704	0.0001	0.474
	[Py41][Tf2N]	0.0042	0.394	0.0198	0.053	-0.0237	0.738	-0.0002	0.535
	[P4441][Tf2N]	-0.0210	0.986	-0.4479	0.993	-0.4330	0.976	-0.0018	0.936
	[N1888][Tf2N]	-0.0346	0.990	-0.6096	0.998	-0.5312	0.981	-0.0037	0.990
Conventional films	TCP	0.0021	0.979	0.0420	1.000	0.0353	0.970	0.0001	0.961
	Apiezon-L	0.0002	0.658	0.0007	0.673	0.0011	0.575	0.0000	0.746
	PEG-1k	0.0009	0.960	0.0090	0.929	0.0066	0.990	0.0000	0.901
	OV-17	0.0000	0.053	0.0035	0.869	0.0015	0.924	0.0000	0.131
	Siponate DS-10	0.0001	0.627	0.0001	0.001	0.0028	0.952	0.0000	0.547

References

1. Freire, M.G.; Teles, A.R.R.; Rocha, M.A.A.; Schröder, B.; Neves, C.M.S.S.; Carvalho, P.J.; Evtuguin, D. V.; Santos, L.M.N.B.F.; Coutinho, J.A.P. Thermophysical characterization of ionic liquids able to dissolve biomass. *J. Chem. Eng. Data* **2011**, *56*, 4813–4822. doi:10.1021/je200790q.
2. Harris, K.R.; Kanakubo, M. Self-diffusion coefficients and related transport properties for a number of fragile ionic liquids. *J. Chem. Eng. Data* **2016**, *61*, 2399–2411. doi:10.1021/acs.jced.6b00021.
3. Tariq, M.; Serro, A.P.; Mata, J.L.; Saramago, B.; Esperança, J.M.S.S.; Canongia Lopes, J.N.; Rebelo, L.P.N. High-temperature surface tension and density measurements of 1-alkyl-3-methylimidazolium bistriflamide ionic liquids. *Fluid Phase Equilib.* **2010**, *294*, 131–138. doi:10.1016/J.FLUID.2010.02.020.
4. Tariq, M.; Carvalho, P.J.; Coutinho, J.A.P.; Marrucho, I.M.; Lopes, J.N.C.; Rebelo, L.P.N. Viscosity of (C2–C14) 1-alkyl-3-methylimidazolium bis(trifluoromethylsulfonyl)amide ionic liquids in an extended temperature range. *Fluid Phase Equilib.* **2011**, *301*, 22–32. doi:10.1016/J.FLUID.2010.10.018.
5. Annat, G.; Forsyth, M.; MacFarlane, D.R. Ionic Liquid Mixtures—Variations in Physical Properties and Their Origins in Molecular Structure. *J. Phys. Chem. B* **2012**, *116*, 8251–8258. doi:10.1021/jp3012602.

6. Neves, C.M.S.S.; Kurnia, K.A.; Coutinho, J.A.P.; Marrucho, I.M.; Lopes, J.N.C.; Freire, M.G.; Rebelo, L.P.N. Systematic study of the thermophysical properties of imidazolium-based ionic liquids with cyano-functionalized anions. *J. Phys. Chem. B* **2013**, *117*, 10271–10283. doi:10.1021/jp405913b.
7. de Castro, C.A.N.; Langa, E.; Morais, A.L.; Lopes, M.L.M.; Lourenço, M.J.V.; Santos, F.J.V.; Santos, M.S.C.S.; Lopes, J.N.C.; Veiga, H.I.M.; Macatrão, M.; et al. Studies on the density, heat capacity, surface tension and infinite dilution diffusion with the ionic liquids [C4mim][NTf2], [C4mim][dca], [C2mim][EtOSO3] and [Aliquat][dca]. *Fluid Phase Equilib.* **2010**, *294*, 157–179. doi:10.1016/j.fluid.2010.03.010.
8. Engelmann, M.; Schmidt, H.; Safarov, J.; Nocke, J.; Hassel, E. Thermal properties of 1-butyl-3-methylimidazolium dicyanamide at high pressures and temperatures. *Acta Chim. Slovaca* **2012**, *5*, 86–94. doi:10.2478/v10188-012-0014-2 Open access.
9. Andanson, J.-M.; Meng, X.; Traïkia, M.; Husson, P. Quantification of the impact of water as an impurity on standard physico-chemical properties of ionic liquids. *J. Chem. Thermodyn.* **2016**, *94*, 169–176. doi:10.1016/J.JCT.2015.11.008.
10. Seoane, R.G.; Corderí, S.; Gómez, E.; Calvar, N.; González, E.J.; Macedo, E.A.; Domínguez, Á. Temperature Dependence and Structural Influence on the Thermophysical Properties of Eleven Commercial Ionic Liquids. *Ind. Eng. Chem. Res.* **2012**, *51*, 2492–2504. doi:10.1021/ie2029255.
11. Safarov, J.; Geppert-Rybczyńska, M.; Kul, I.; Hassel, E. Thermophysical properties of 1-butyl-3-methylimidazolium acetate over a wide range of temperatures and pressures. *Fluid Phase Equilib.* **2014**, *383*, 144–155. doi:10.1016/j.fluid.2014.10.015.
12. Almeida, H.F.D.; Passos, H.; Lopes-da-Silva, J.A.; Fernandes, A.M.; Freire, M.G.; Coutinho, J.A.P. Thermophysical Properties of Five Acetate-Based Ionic Liquids. *J. Chem. Eng. Data* **2012**, *57*, 3005–3013. doi:10.1021/je300487n.
13. Govinda, V.; Attri, P.; Venkatesu, P.; Venkateswarlu, P. Thermophysical properties of dimethylsulfoxide with ionic liquids at various temperatures. *Fluid Phase Equilib.* **2011**, *304*, 35–43. doi:10.1016/j.fluid.2011.02.010.
14. Seddon, K.R.; Stark, A.; Torres, M.J. Viscosity and density of 1-alkyl-3-methylimidazolium ionic liquids. *ACS Symp. Ser.* **2002**, *819*, 34–49. doi:10.1021/bk-2002-0819.ch004.
15. Salgado, J.; Regueira, T.; Lugo, L.; Vijande, J.; Fernández, J.; García, J. Density and viscosity of three (2,2,2-trifluoroethanol + 1-butyl-3-methylimidazolium) ionic liquid binary systems. *J. Chem. Thermodyn.* **2014**, *70*, 101–110. doi:10.1016/J.JCT.2013.10.027.
16. Kim, K.-S.; Shin, B.-K.; Lee, H. Physical and electrochemical properties of 1-butyl-3-methylimidazolium bromide, 1-butyl-3-methylimidazolium iodide, and 1-butyl-3-methylimidazolium tetrafluoroborate. *Korean J. Chem. Eng.* **2004**, *21*, 1010–1014. doi:10.1007/BF02705586.
17. Izgorodina, E.I.; Maganti, R.; Armel, V.; Dean, P.M.; Pringle, J.M.; Seddon, K.R.; MacFarlane, D.R. Understanding the Effect of the C2 Proton in Promoting Low Viscosities and High Conductivities in Imidazolium-Based Ionic Liquids: Part I. Weakly Coordinating Anions. *J. Phys. Chem. B* **2011**, *115*, 14688–14697. doi:10.1021/jp208573y.
18. Kandil, M.E.; Marsh, K.N.; Goodwin, A.R.H. Measurement of the viscosity, density, and electrical conductivity of 1-hexyl-3-methylimidazolium bis(trifluorosulfonyl)imide at temperatures between (288 and 433) K and pressures below 50 MPa. *J. Chem. Eng. Data* **2007**, *52*, 2382–2387. doi:10.1021/je7003484.
19. Klomfar, J.; Součková, M.; Pátek, J. Temperature Dependence Measurements of the Density at 0.1 MPa for 1-Alkyl-3-methylimidazolium-Based Ionic Liquids with the Trifluoromethanesulfonate and Tetrafluoroborate Anion. *J. Chem. Eng. Data* **2010**, *55*, 4054–4057. doi:10.1021/je100185e.
20. Vakili-Nezhaad, G.; Vatani, M.; Asghari, M.; Ashour, I. Effect of temperature on the physical properties of 1-butyl-3-methylimidazolium based ionic liquids with thiocyanate and tetrafluoroborate anions, and 1-hexyl-3-methylimidazolium with tetrafluoroborate and hexafluorophosphate anions. *J. Chem. Thermodyn.* **2012**, *54*, 148–154. doi:10.1016/J.JCT.2012.03.024.
21. Li, J.-G.; Hu, Y.-F.; Sun, S.-F.; Liu, Y.-S.; Liu, Z.-C. Densities and dynamic viscosities of the binary system (water + 1-hexyl-3-methylimidazolium bromide) at different temperatures. *J. Chem. Thermodyn.* **2010**, *42*, 904–908. doi:10.1016/J.JCT.2010.03.002.
22. Mac Dowell, N.; Llovell, F.; Sun, N.; Hallett, J.P.; George, A.; Hunt, P.A.; Welton, T.; Simmons, B.A.; Vega, L.F. New experimental density data and soft-SAFT models of alkylimidazolium ([CnC1im]+) chloride (Cl-), methylsulfate ([MeSO4]-), and dimethylphosphate ([Me 2PO4]-) based ionic liquids. *J. Phys. Chem. B* **2014**, *118*, 6206–6221. doi:10.1021/jp501619y.

23. Malek, N.I.; Singh, A.; Surati, R.; Ijardar, S.P. Study on thermo physical and excess molar properties of binary systems of ionic liquids. I: [Cnmim][PF6] (n = 6, 8) and alkyl acetates. *J. Chem. Thermodyn.* **2014**, *74*, 103–118. doi:10.1016/J.JCT.2014.01.012.
24. Safarov, J.; Bussemer, C.; Aliyev, A.; Lafuente, C.; Hassel, E.; Abdulagatov, I. Effect of temperature on thermal (density), caloric (heat capacity), acoustic (speed of sound) and transport (viscosity) properties of 1-octyl-3-methylimidazolium hexafluorophosphate at atmospheric pressure. *J. Chem. Thermodyn.* **2018**, *124*, 49–64. doi:10.1016/J.JCT.2018.04.018.
25. Carrera, G.V.S.M.; Afonso, C.A.M.; Branco, L.C. Interfacial properties, densities, and contact angles of task specific ionic liquids *J. Chem. Eng. Data* **2010**, *55*, 609–615. doi:10.1021/je900502s.
26. Bhattacharjee, A.; Carvalho, P.J.; Coutinho, J.A.P. The effect of the cation aromaticity upon the thermophysical properties of piperidinium- and pyridinium-based ionic liquids. *Fluid Phase Equilib.* **2014**, *375*, 80–88. doi:10.1016/j.fluid.2014.04.029.
27. Rodil, E.; Arce, A.; Arce, A.; Soto, A. Measurements of the density, refractive index, electrical conductivity, thermal conductivity and dynamic viscosity for tributylmethylphosphonium and methylsulfate based ionic liquids. *Thermochim. Acta* **2018**, *664*, 81–90. doi:10.1016/j.tca.2018.04.007.
28. Gaciño, F.M.; Regueira, T.; Lugo, L.; Comuñas, M.J.P.; Fernández, J. Influence of molecular structure on densities and viscosities of several ionic liquids. *J. Chem. Eng. Data* **2011**, *56*, 4984–4999. doi:10.1021/je200883w.
29. Bhattacharjee, A.; Luís, A.; Santos, J.H.; Lopes-da-Silva, J.A.; Freire, M.G.; Carvalho, P.J.; Coutinho, J.A.P. Thermophysical properties of sulfonium- and ammonium-based ionic liquids. *Fluid Phase Equilib.* **2014**, *381*, 36–45. doi:10.1016/J.FLUID.2014.08.005.
30. Fröba, A.P.; Kremer, H.; Leipertz, A. Density, refractive index, interfacial tension, and viscosity of ionic liquids [EMIM][EtSO4], [EMIM][NTf2], [EMIM][N(CN) 2], and [OMA][NTf2] in dependence on temperature at atmospheric pressure. *J. Phys. Chem. B* **2008**, *112*, 12420–12430. doi:10.1021/jp804319a.
31. Toxicological risks of selected flame-retardant chemicals. In *National Research Council (U.S.). Subcommittee on Flame-Retardant Chemicals*; National Academic Press: Washington, DC, USA. ISBN 0309070473.
32. Apiezon Apiezon L, M & N GREASES. Available online: https://static.mimaterials.com/apiezon/DocumentLibrary/TechnicalDatasheets/Apiezon_L_M_and_N_Ultra_High_and_High_Vacuum_Greases_Datasheet.pdf (accessed on 20 July 2020)
33. Polyethylene glycol - MAK Value Documentation -1998. In *The MAK-Collection for Occupational Health and Safety*; Wiley-VCH Verlag GmbH & Co. KGaA: Weinheim, Germany, 2002; pp. 248–270. doi:10.1002/3527600418.mb2532268kske0010.
34. Trivedi, S.; Pandey, S. Interactions within a [ionic liquid + poly(ethylene glycol)] mixture revealed by temperature-dependent synergistic dynamic viscosity and probe-reported microviscosity. *J. Phys. Chem. B* **2011**, *115*, 7405–7416. doi:10.1021/jp203079p.
35. Silicone oil (high temperature) | 63148-58-3 Properties Available online: https://www.chemicalbook.com/ChemicalProductProperty_EN_CB3189461.htm (accessed on 6 September 2019).
36. Ohio Valley Specialty *Gas Chromatography Supplies Catalog 62*; 2013;
37. Sodium dodecylbenzenesulfonate - Registration Dossier - ECHA Available online: https://echa.europa.eu/registration-dossier/-/registered-dossier/11639/4/23 (accessed on 6 September 2019).
